# Kinetics of Riboflavin Production by *Hyphopichia wangnamkhiaoensis* under Varying Nutritional Conditions

**DOI:** 10.3390/ijms25179430

**Published:** 2024-08-30

**Authors:** Raziel Arturo Jiménez-Nava, Griselda Ma. Chávez-Camarillo, Eliseo Cristiani-Urbina

**Affiliations:** 1 Departamento de Ingeniería Bioquímica, Escuela Nacional de Ciencias Biológicas, Instituto Politécnico Nacional, Avenida Wilfrido Massieu s/n, Unidad Profesional Adolfo López Mateos, Mexico City 07738, Mexico; 2 Departamento de Microbiología, Escuela Nacional de Ciencias Biológicas, Instituto Politécnico Nacional, Prolongación de Carpio y Plan de Ayala s/n, Colonia Santo Tomás, Mexico City 11340, Mexico

**Keywords:** batch culture, *Hyphopichia wangnamkhiaoensis*, nutritional factors, pneumatic bioreactor, riboflavin, vitamin B_2_, yeast

## Abstract

Riboflavin, an essential vitamin for humans, is extensively used in various industries, with its global demand being met through fermentative processes. *Hyphopichia wangnamkhiaoensis* is a novel dimorphic yeast species capable of producing riboflavin. However, the nutritional factors affecting riboflavin production in this yeast species remain unknown. Therefore, we conducted a kinetic study on the effects of various nutritional factors—carbon and energy sources, nitrogen sources, vitamins, and amino acids—on batch riboflavin production by *H. wangnamkhiaoensis*. Batch experiments were performed in a bubble column bioreactor to evaluate cell growth, substrate consumption, and riboflavin production. The highest riboflavin production was obtained when the yeast growth medium was supplemented with glucose, ammonium sulfate, biotin, and glycine. Using these chemical components, along with the mineral salts from Castañeda-Agullo’s culture medium, we formulated a novel, low-cost, and effective culture medium (the RGE medium) for riboflavin production by *H. wangnamkhiaoensis*. This medium resulted in the highest levels of riboflavin production and volumetric productivity, reaching 16.68 mg/L and 0.713 mg/L·h, respectively, within 21 h of incubation. These findings suggest that *H. wangnamkhiaoensis*, with its shorter incubation time, could improve the efficiency and cost-effectiveness of industrial riboflavin production, paving the way for more sustainable production methods.

## 1. Introduction

Riboflavin (vitamin B_2_) is essential for humans and other animals. Healthy, well-nourished adults require 0.9–1.3 mg of riboflavin daily, which must be obtained from foods (meat, eggs, milk, dairy products, beef liver, broccoli, turnip, soybeans, peanuts, etc.) or supplements, with any excess excreted in urine [1,2,3,4].

Riboflavin primarily functions as a precursor to the coenzymes flavin adenine dinucleotide (FAD) and flavin mononucleotide (FMN), which serve as electron carriers in many critical biological oxidation-reduction reactions [1,2,3]. Additionally, riboflavin is a key component of blue light-sensitive cryptochrome, which plays a role in synchronizing circadian rhythms and sensing magnetic fields in several species [5].

Riboflavin is extensively used in the feed, food, pharmaceutical, and cosmetic industries [1,2,3,6]. In 2020, the global sales of riboflavin exceeded USD 250 million, with an annual production of 10,000 tons [3]. Of this, only approximately 10% is used in drug and multivitamin formulations, 10–20% is used as a colorant, and the majority is used as animal feed [3,7,8].

The global riboflavin demand has been fulfilled biotechnologically, with production exclusively achieved by microbial fermentation. *Bacillus subtilis*, *Ashbya gossypii*, and *Candida famata* have been the primary production models in recent decades [1,2,6,9,10]. Nevertheless, various other micro-organisms, including bacteria, filamentous fungi, and yeasts, are also promising candidates for riboflavin overproduction [1,2,6,9,10,11,12,13,14,15].

Yeasts are well known for their ability to biosynthesize sufficient riboflavin for their growth, with some species even overproducing and excreting riboflavin into the fermentation broth [16]. They offer several advantages for large-scale production because of their high specific growth rate, short duplication time, ease of cultivation and handling, metabolic versatility, low maintenance requirements, safety, and ease of genetic modification [13,17]. Additionally, downstream processing for riboflavin separation and purification is more straightforward, less costly, and less technologically challenging because of the simple chemical composition and rheological properties of the culture media used for their growth and riboflavin production [8,18].

Among flavinogenic yeasts, the wild-type and recombinant strains of *Meyerozyma guilliermondii* and *Candida famata* have been the most extensively studied for riboflavin production, with the latter being used for large-scale industrial production [1,3,6,13,18,19,20,21,22,23,24,25,26,27,28,29,30].

Although microbial riboflavin biosynthesis is a highly specific metabolic process, the metabolic flux to riboflavin is influenced by many non-specific reactions [6,31,32]. This presents an opportunity to enhance riboflavin production through the modification of environmental and nutritional conditions for the growth of riboflavin-producing micro-organisms. In this context, the composition of the culture medium is a crucial factor affecting cell growth, riboflavin production, and the costs associated with riboflavin separation and purification [7,8,9,33].

*Hyphopichia wangnamkhiaoensis* is a novel dimorphic yeast species known for its remarkable capacity to produce α-amylase [17,34,35] and oleic acid [36]. Recently, this yeast species was reported to excrete a brilliant yellow, fluorescent compound when grown in a yeast nitrogen base (YNB) medium supplemented with glucose [37]. This compound was isolated using a reverse-phase high-performance liquid chromatography–diode array detector and identified as riboflavin through proton nuclear magnetic resonance (^1^H NMR), UV-Vis, and fluorescence spectroscopy [38].

However, to date, no reports exist in the literature regarding the nutritional factors affecting riboflavin production by this yeast species. Therefore, the primary aim of this study was to kinetically evaluate the influence of different nutritional factors (carbon sources, nitrogen sources, amino acids, and vitamins) on the growth, substrate consumption, and riboflavin production in batch cultures of *H. wangnamkhiaoensis*. The effects of different carbon and energy sources, nitrogen sources, amino acids, and vitamins were investigated using a yeast nitrogen base (YNB), yeast nitrogen base without amino acids and ammonium sulfate (YNB-w/o-N), YNB-w/o-N, and yeast nitrogen base without vitamins (YNB-VF) as basal media, respectively. These culture media were initially used as basal media because previous studies have shown that *H. wangnamkhiaoensis* can produce riboflavin in these media. For this purpose, a monothetic analysis, also known as the one-factor-at-a-time (OFAT) method or the one-variable-at-a-time method, where one factor or variable is studied independently while keeping the others constant [39], was used in this study. This method is helpful when no information is available on the effects of possible factors on the object of study [40]. This method also allows for an accurate understanding of the effects of each factor [39]. For microbiological culture media optimization, OFAT provides insights into crucial physical and nutritional factors and helps in the design of culture medium formulations [41,42].

As the filamentous cells of the dimorphic yeast *H. wangnamkhiaoensis* are damaged by the shear stress generated in stirred bioreactors, leading to reduced growth rates and lower metabolite production [17], a bubble column bioreactor was employed in this study to minimize cell damage, enhance riboflavin production, and reduce production costs. Additionally, we aimed to formulate an alternative, simple, and low-cost culture medium to produce riboflavin using this yeast species.

## 2. Results and Discussion

### 2.1. Effect of Various Carbon Sources on Riboflavin Production by H. wangnamkhiaoensis in a Bubble Column Bioreactor

*Hyphopichia wangnamkhiaoensis*, formerly known as *Wickerhamia* sp. [34,35] and *Candida wangnamkhiaoensis* [17,36,43], is an anamorphic yeast capable of assimilating a wide variety of carbon sources [43], with glucose, maltose, starch, and glycerol being the most suitable for cell growth [17,34,35,36]. Therefore, in the present study, these carbon sources were assayed for biomass and riboflavin production by *H. wangnamkhiaoensis*.

**Figure 1:**
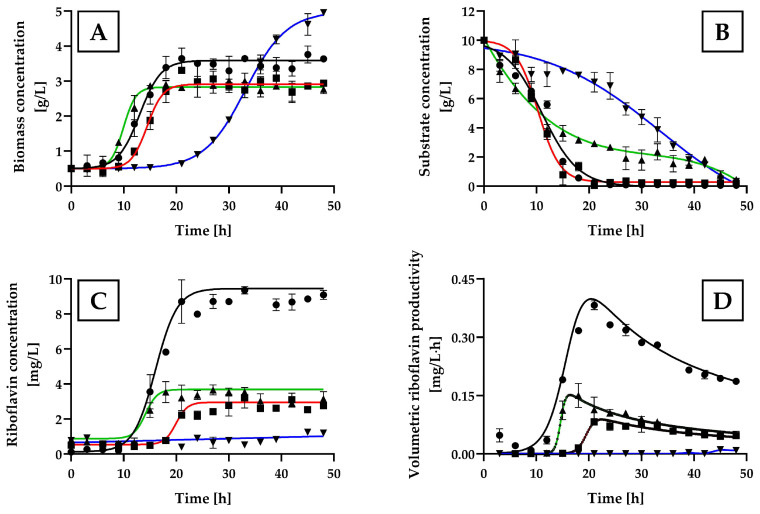
Kinetic profiles of cell growth, substrate consumption, and riboflavin production by batch cultures of *Hyphopichia wangnamkhiaoensis* in yeast nitrogen base (YNB) medium supplemented with various carbon sources. Experiments were conducted in a bubble column bioreactor at 28 ± 1 °C, pH 5.6, and 1 vvm of air flow rate. The carbon sources used in the study are represented by the following symbols: (●) glucose, (■) maltose, (▲) starch, and (▼) glycerol. The profiles include (**A**) biomass concentration, (**B**) substrate concentration, (**C**) riboflavin concentration, and (**D**) volumetric riboflavin productivity. Error bars represent the standard deviation of independent triplicates. When the error bars are not shown, the standard deviation is smaller than the symbol size.

The kinetic patterns of cell growth, substrate consumption, and riboflavin production are shown in Figure 1.

Despite a longer lag phase for *H. wangnamkhiaoensis* with glycerol compared to the other carbon sources tested, the highest biomass concentration (4.98 ± 0.12 g/L) was achieved with glycerol, followed by glucose (3.57 ± 0.21 g/L), maltose (2.93 ± 0.32 g/L), and starch (2.89 ± 0.17 g/L), after 48 h of incubation (Figure 1A). These results are consistent with the 3.35 g biomass/L obtained by Pérez-Rodríguez et al. in flask-level batch cultures using glucose [36] but not with glycerol, where their reported value was 3.6 g/L compared to 4.98 g/L in this study. These differences may be attributed to differences in the culture medium composition, culture conditions, and cultivation vessels (flask vs. bubble column reactor).

Glycerol typically exhibits a low uptake rate in yeasts, which severely limits its effectiveness as a carbon and energy source for growth [44]. However, the *H. wangnamkhiaoensis* yeast species used in this study achieved a higher cell concentration with glycerol in a bubble column bioreactor compared to other more readily assimilated carbon sources such as glucose and maltose (*p* < 0.05).

The substrate consumption patterns aligned with the growth patterns observed. Glucose was consumed more rapidly than maltose, starch, or glycerol. Over a 24 h incubation period, glucose was nearly depleted, with a residual concentration of 0.09 ± 0.02 g/L, consistent with previous findings for this yeast species [36]. While maltose consumption ceased at 30 h of incubation, reaching a residual concentration of 0.23 ± 0.03 g/L, starch and glycerol consumption continued throughout the 48 h experiment, with final residual concentrations of 0.46 ± 0.21 g/L and 0.32 ± 0.10 g/L, respectively (Figure 1B).

Among the carbon and energy sources assayed, glucose was the most effective for riboflavin production by *H. wangnamkhiaoensis*, achieving a concentration of 8.88 ± 0.42 mg of riboflavin/L (Figure 1C). This was statistically significantly higher [as determined by one-way analysis of variance (ANOVA) with Bonferroni’s test] compared to the riboflavin concentrations obtained with starch (3.32 ± 0.42 mg/L), maltose (2.65 ± 0.41 mg/L) and glycerol (1.22 ± 0.05 mg/L) (*p* < 0.05). These findings indicate that, despite glycerol resulting in higher biomass production than glucose, maltose, or starch, it was not an effective carbon source for riboflavin production by *H. wangnamkhiaoensis*.

Figure 1D shows the kinetic profiles of volumetric riboflavin productivity for the different carbon sources assayed. Glucose resulted in the highest volumetric riboflavin productivity, reaching a value of 0.37 ± 0.08 mg/L·h at 21 h of incubation. This value is statistically significantly higher (*p* < 0.05) compared to the maximum volumetric productivity achieved with starch (0.15 ± 0.03 mg/L·h), maltose (0.08 ± 0.01 mg/L·h), and glycerol (0.01 ± 0.003 mg/L·h).

These results clearly indicate that *H. wangnamkhiaoensis* prefers glucose over other carbon sources for riboflavin production. Therefore, glucose was selected as the carbon and energy source for subsequent experiments.

Previous studies have shown that glucose enhances riboflavin production in flavinogenic yeast strains [6,12,18,20,25,33,45,46]. This is because guanosine triphosphate (GTP) and ribulose-5-phosphate (Ru5P), which are derived from the purine biosynthetic pathway (PBP) and pentose phosphate pathway (PPP), respectively, are key riboflavin biosynthesis precursors [2,6,25,47] that are synthesized from glucose [6,48,49,50]. Other carbon sources that flavinogenic yeasts prefer for riboflavin production include sucrose and fructose, rather than maltose or galactose [31,33].

### 2.2. Effect of Various Nitrogen Sources on Riboflavin Production by H. wangnamkhiaoensis

#### 2.2.1. Flask Screening of Nitrogen Sources for Riboflavin Production by *H. wangnamkhiaoensis*

*Hyphopichia wangnamkhiaoensis* is a novel yeast species that has rarely been studied, leaving its physiological and metabolic capacities largely unknown. Consequently, there is limited information on the nitrogen source assimilation ability of this yeast species [43]. To address this, a nitrogen source assimilation test—using (NH_4_Cl, (NH_4_)_2_SO_4_, (NH_4_)_2_HPO_4_, NH_4_CH_3_CO_2_, urea, NaNO_3_, NaNO_2_, KNO_3_, L-glutamic acid, and DL-glutamic acid—was conducted following the liquid assimilation assays described by Yarrow [51] and Wickerham [52]. Additionally, a preliminary screening of nitrogen sources for riboflavin production was performed in Erlenmeyer flasks using a YNB-w/o-N basal medium supplemented with glucose as the carbon source, at 28 ± 1 °C, pH 5.6, with an agitation rate of 120 rpm, over 72 h.

*Hyphopichia wangnamkhiaoensis* grew with all the tested nitrogen sources except NaNO_2_. However, its growth was weak when NaNO_3_ and KNO_3_ were used. Limtong et al. [43] reported that *Candida wangnamkhiaoensis*, now called *H. wangnamkhiaoensis*, cannot use KNO_3_ as a nitrogen source for growth, in contrast to the present results. This discrepancy may be due to differences in chemical compositions of the culture media used in the assays.

**Figure 2:**
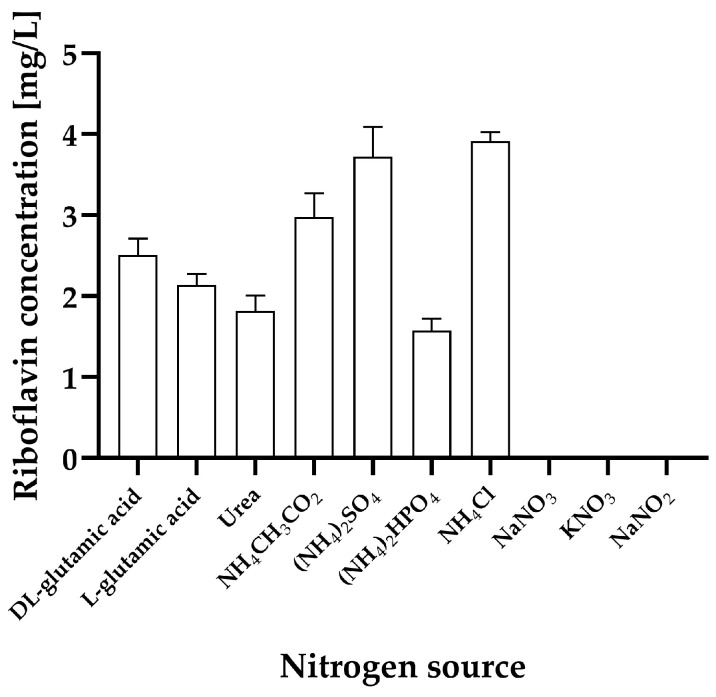
Effect of various nitrogen sources on riboflavin production by the yeast *Hyphopichia wangnamkhiaoensis* at flask level, using yeast nitrogen base without amino acids and ammonium sulfate (YNB-w/o-N) at 28 ± 1 °C and pH 5.6. The nitrogen sources tested included DL-glutamic acid, L-glutamic acid, urea, NH_4_CH_3_CO_2_, (NH_4_)_2_SO_4_, (NH_4_)_2_HPO_4_, NH_4_Cl, NaNO_3_, KNO_3_, and NaNO_2_, with riboflavin concentrations measured after 72 h of incubation. Error bars represent the standard deviation of independent triplicates.

Although *H. wangnamkhiaoensis* grew with almost all the nitrogen sources assayed, it produced riboflavin only with the following nitrogen sources (in descending order): NH_4_Cl (3.91 ± 0.12 mg/L), (NH_4_)_2_SO_4_ (3.72 ± 0.37 mg/L), NH_4_CH_3_CO_2_ (2.98 ± 0.29 mg/L), DL-glutamic acid (2.51 ± 0.20 mg/L), L-glutamic acid (2.14 ± 0.13 mg/L), urea (1.82 ± 0.1 mg/L), and (NH_4_)_2_HPO_4_ (1.57 ± 0.15 mg/L) (Figure 2). No statistically significant differences were observed in riboflavin production when NH_4_Cl and (NH_4_)_2_SO_4_ were used as nitrogen sources (*p* > 0.05). However, there were statistically significant differences in riboflavin production between the nitrogen sources NH_4_Cl and (NH_4_)_2_SO_4_ and the others tested (*p* < 0.05). Similarly, significant statistical differences were found between NH_4_CH_3_CO_2_ and the other nitrogen sources (*p* < 0.05).

These results clearly show that NH_4_^+^ salts (NH_4_Cl, (NH_4_)_2_SO_4_, and NH_4_CH_3_CO_2_) are the most effective nitrogen sources for riboflavin production by *H. wangnamkhiaoensis*. Consequently, these salts were selected for kinetic studies of cell growth, substrate consumption, and riboflavin production in the bubble column reactor.

These results may be attributed to the incorporation of NH_4_^+^ ions into amino acids, such as glutamine [53,54], glycine [6,53,55,56], and aspartate [53,54], which are essential for synthesizing GTP, a precursor of riboflavin. Similarly, some studies have shown improved NH_4_^+^ ion assimilation when yeasts were grown on glucose as a carbon source [57].

#### 2.2.2. Effect of the Selected Nitrogen Sources on Cell Growth, Glucose Consumption, and Riboflavin Production by *H. wangnamkhiaoensis* in a Bubble Column Bioreactor

**Figure 3:**
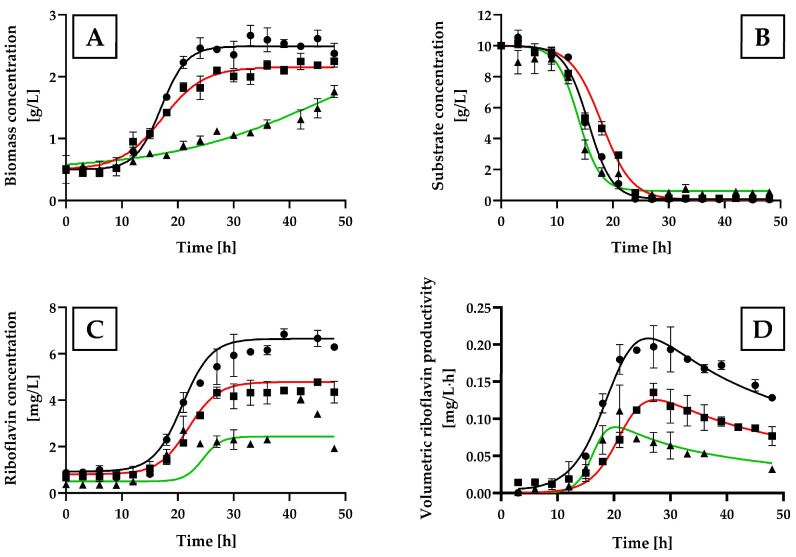
Kinetic profiles of cell growth, glucose consumption, and riboflavin production by *Hyphophichia wangnamkhiaoensis* in various nitrogen sources. Experiments were conducted in a bubble column reactor using the yeast nitrogen base without amino acids and ammonium sulfate (YNB-w/o-N) medium supplemented with different nitrogen sources, represented by the following symbols: (●) (NH_4_)_2_SO_4_, (■) NH_4_Cl, and (▲) NH_4_CH_3_CO_2_, at 28 ± 1 °C, pH 5.6, and 1 vvm of air flow rate. The profiles include (**A**) biomass concentration, (**B**) glucose concentration, (**C**) riboflavin concentration, and (**D**) volumetric riboflavin productivity. Error bars represent the standard deviation of independent triplicates. When the error bars are not shown, the standard deviation is smaller than the symbol size.

Figure 3A illustrates that the growth curves of *H. wangnamkhiaoensis* cultivated with (NH_4_)_2_SO_4_ and NH_4_Cl were comparable, both showing similar lag phases (approximately 9 h) and similar durations to reach the stationary growth phase (24 h). Conversely, yeast grown in NH_4_CH_3_CO_2_ exhibited an extended lag phase and did not reach a stationary growth phase during the entire incubation period.

(NH_4_)_2_SO_4_ yielded the highest *H. wangnamkhiaoensis* biomass production (2.48 ± 0.30 g/L), followed by NH_4_Cl (2.09 ± 0.27 g/L), and NH_4_CH_3_CO_2_ (1.83 ± 0.38 g/L) (*p* < 0.05) (Figure 3A).

During the experiment in the bubble column bioreactor, the concentration of glucose in the yeast cultures decreased gradually over time until it was utterly exhausted at approximately 24 h of incubation when (NH_4_)_2_SO_4_ and NH_4_Cl were used as nitrogen sources. In contrast, the residual glucose concentration in the yeast culture supplemented with NH_4_CH_3_CO_2_ remained nearly constant at 0.51 ± 0.11 g/L even after 24 h of incubation (Figure 3B).

The kinetic profiles of riboflavin production by *H. wangnamkhiaoensis* indicated that the highest riboflavin levels were achieved using (NH_4_)_2_SO_4_ as the nitrogen source, reaching a maximum concentration of 6.40 ± 0.36 g/L (Figure 3C). This value was statistically significantly higher than the values obtained with NH_4_Cl (4.38 ± 0.35 g/L) and NH_4_CH_3_CO_2_ (2.66 ± 0.72 mg/L) (*p* < 0.05).

The riboflavin concentrations achieved with (NH_4_)_2_SO_4_ and NH_4_Cl in the bubble column bioreactor were higher than those achieved in the flask (*p* < 0.05), which may be attributed to the improved hydrodynamic and mass-transfer characteristics of the bubble column bioreactor.

The variation in the volumetric productivity of riboflavin as a function of incubation time for the different nitrogen sources assayed is shown in Figure 3D. The highest volumetric productivity of riboflavin was attained at 27 h of incubation using (NH_4_)_2_SO_4_ as a nitrogen source, with a value of 0.20 ± 0.03 mg/L·h. This was significantly higher than the volumetric productivity achieved with NH_4_Cl (0.14 ± 0.01 mg/L·h at 27 h) and NH_4_CH_3_CO_2_ (0.11 ± 0.03 mg/L·h at 21 h) (*p* < 0.05).

These results clearly showed that (NH_4_)_2_SO_4_ yielded the best biomass and riboflavin production characteristics for *H. wangnamkhiaoensis*; therefore, this nitrogen source was used in subsequent experiments.

(NH_4_)_2_SO_4_ has been the most commonly used nitrogen source in flavinogenic yeast research, with concentrations ranging from 5 to 10 g/L or 0.75 g of nitrogen/L [45]. Similarly, yeast extract peptone dextrose (YPD), YNB, and Burkholder’s culture media are the most preferred culture media for flavinogenic yeast species, with the latter two containing (NH_4_)_2_SO_4_. Furthermore, Burkholder’s culture medium has been supplemented with yeast extract and (NH_4_)_2_SO_4_ at concentrations ranging from 2 g/L to 5 g/L [18,20,25,58].

### 2.3. Effect of Histidine, Methionine, and Tryptophan on Cell Growth, Glucose Consumption, and Riboflavin Production by H. wangnamkhiaoensis in a Bubble Column Bioreactor

As the YNB culture medium contains DL-threonine, L-histidine, and DL-tryptophan, we aimed to evaluate the individual effects of these amino acids on batch riboflavin production by *H. wangnamkhiaoensis*. The kinetic experiments were conducted in a bubble column bioreactor using the YNB-w/o-N culture medium supplemented with glucose (10 g/L), (NH_4_)_2_SO_4_ (5 g/L), and one of the test amino acids (histidine, methionine, or tryptophan). As a negative control (amino acid-free control), we also analyzed *H. wangnamkhiaoensis* cells cultured in YNB-w/o-N medium supplemented with glucose (10 g/L) and (NH_4_)_2_SO_4_ (5 g/L) but without added amino acids.

**Figure 4:**
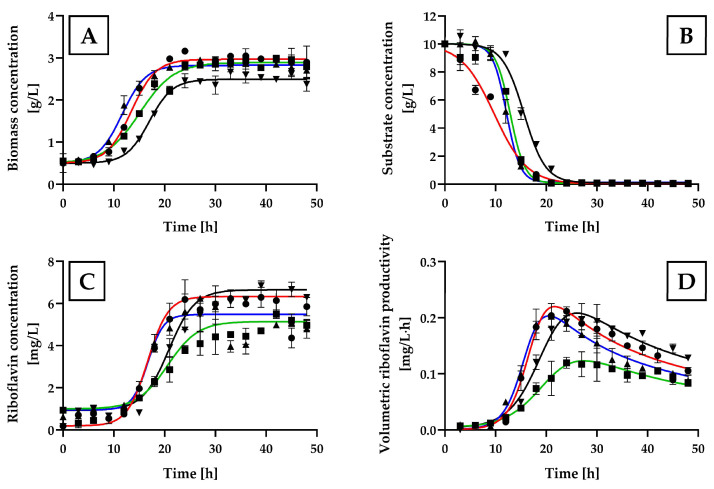
Effect of amino acid supplementation on the kinetic profiles of cell growth, glucose consumption, and riboflavin production by *Hyphopichia wangnamkhiaoensis*. Experiments were conducted in a bubble column reactor using different culture media, represented by the following symbols: (▼) yeast nitrogen base without amino acids and ammonium sulfate (YNB-w/o-N) medium (control culture without amino acids), and YNB-w/o-N medium supplemented with (●) L-histidine, (■) DL-methionine, and (▲) DL-tryptophan in a bubble column reactor, at 28 ± 1 °C, pH 5.6, and 1 vvm of air flow rate. The kinetic profiles shown include (**A**) biomass concentration, (**B**) glucose concentration, (**C**) riboflavin concentration, and (**D**) volumetric productivity of riboflavin. Error bars represent the standard deviation of independent triplicates. When the error bars are not shown, the standard deviation is smaller than the symbol size.

The growth curves were similar across all yeast cultures assayed; however, the maximum biomass concentration in cultures supplemented with amino acids was slightly higher than that in the control without amino acids. In addition, no statistically significant difference in the biomass concentration was observed among cultures supplemented with L-histidine (2.97 ± 0.20 g/L), DL-methionine (2.88 ± 0.13 g/L), or DL-tryptophan (2.83 ± 0.12 g/L) (*p* > 0.05); however, the biomass concentration with these amino acids was significantly higher compared to the control culture (2.48 ± 0.30 g/L) (*p* < 0.05) (Figure 4A).

The glucose consumption patterns in yeast cultures treated with DL-methionine and DL-tryptophan were nearly identical (*p* > 0.05). In contrast, the patterns for the control culture and the culture supplemented with L-histidine showed slight differences. However, glucose was completely consumed by all yeast cultures within approximately 21–24 h of incubation (Figure 4B).

The highest riboflavin production was observed in the yeast control culture (6.40 ± 0.36 mg/L) and in the culture supplemented with L-histidine (5.86 ± 0.70 mg/L), with no significant statistical difference between them (*p* > 0.05) (Figure 4C). In contrast, riboflavin production was lower in cultures supplemented with DL-methionine (4.96 ± 0.44 mg/L) and DL-tryptophan (4.79 ± 0.71 mg/L). This variation could be attributed to the effect of the added amino acids on the cellular metabolism of *H. wangnamkhiaoensis*, as confirmed by the yields of riboflavin (Y_PS_) and biomass (Y_XS_) relative to the glucose consumed. The Y_PS_ obtained in the control culture was 0.64 ± 0.04 mg riboflavin/g of glucose, which was approximately 8.47%, 28%, and 33.3% higher than those achieved in the yeast cultures supplemented with L-histidine, DL- methionine, and DL-tryptophan, respectively. This indicates that the yeast converted the consumed glucose to riboflavin more efficiently in the control culture than in the cultures supplemented with any amino acid. Similarly, the Y_XS_ obtained in the control culture (0.20 ± 0.03 g of biomass/g of glucose) was 25%, 17%, and 16% lower than those achieved in the cultures supplemented with L-histidine, DL-methionine, and DL-tryptophan, respectively. This suggests that the addition of any of these amino acids to the yeast growth medium caused more glucose to be utilized for biomass formation rather than riboflavin production, compared to the control culture without amino acids.

However, the volumetric productivity profiles of riboflavin show that the highest productivity values were obtained in the control culture (0.20 ± 0.03 mg/L·h) and in the cultures supplemented with L-histidine (0.21 ± 0.01 mg/L·h) and DL-tryptophan (0.21 ± 0.01 mg/L·h), with no statistical difference between them (*p* > 0.05) (Figure 4D). In contrast, the volumetric riboflavin productivity in the culture supplemented with DL-methionine was the lowest (0.12 ± 0.01 mg/L·h) and statistically different from that in the other cultures (*p* < 0.05).

The above results show that adding any of the amino acids present in the YNB medium (L-histidine, DL-methionine, and DL-tryptophan) to the yeast growth medium did not favor riboflavin production by *H. wangnamkhiaoensis*. Therefore, subsequent experiments were conducted without the addition of any of these three amino acids.

### 2.4. Effect of Various Vitamins on Cell Growth, Glucose Consumption, and Riboflavin Production by H. wangnamkhiaoensis in a Bubble Column Bioreactor

As the YNB and YNB-w/o-N media contain nine vitamins (biotin, calcium pantothenate, folic acid, inositol, niacin, riboflavin, *p*-aminobenzoic acid, pyridoxine hydrochloride, and thiamine hydrochloride), we investigated whether any of these vitamins, except riboflavin, influenced riboflavin production by *H. wangnamkhiaoensis*. Each vitamin was assayed individually. Additionally, a negative control culture using vitamin-free YNB (YNB-VF) medium and a positive control culture using YNB medium (containing all nine vitamins) supplemented with glucose and (NH_4_)_2_SO_4_ were assayed. Table 1 shows the maximum increase in cell biomass (ΔX_max_), maximum specific growth rate (μ_max_), maximum biomass productivity (Rx_max_), overall glucose consumption efficiency (E), maximum riboflavin production (P_max_), and maximum volumetric riboflavin productivity (Rp_max_) of the yeast cultures supplemented with a vitamin and the positive and negative control cultures.

**Figure 5:**
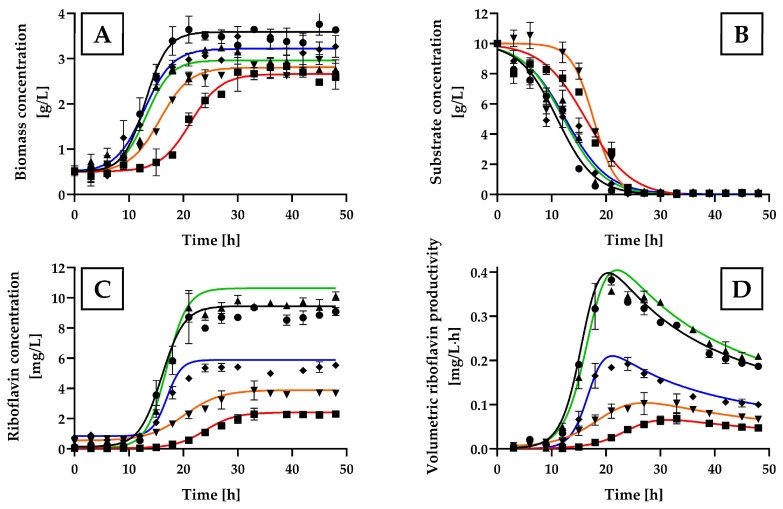
Kinetic profiles of the effect of different vitamins on cell growth, glucose consumption, and riboflavin production by *Hyphopichia wangnamkhiaoensis*. Experiments were conducted in a bubble column bioreactor using different media, represented by the following symbols: (●) yeast nitrogen base medium containing nine vitamins (YNB), (■) vitamin-free YNB (YNB-VF), and YNB-VF medium supplemented with (▲) biotin, (▼) niacin, and (♦) *p*-aminobenzoic acid, at 28 ± 1 °C, pH 5.6, and 1 vvm of air flow rate. The kinetic profiles shown include (**A**) biomass concentration, (**B**) glucose concentration, (**C**) riboflavin concentration, and (**D**) volumetric riboflavin productivity. Error bars represent the standard deviation of independent triplicates. When the error bars are not shown, the standard deviation is smaller than the symbol size.

Similarly, Figure 5 displays the kinetic profiles of cell growth, glucose consumption, and riboflavin production by *H. wangnamkhiaoensis* cultures supplemented with the vitamins that yielded the best performance for riboflavin production and those of the positive and negative control cultures.

The tested vitamins had varying effects on the growth of *H. wangnamkhiaoensis* (Table 1, Figure 5A). Inositol led to the highest increase in cell biomass concentration (3.24 ± 0.20 g/L), followed by YNB (2.91 ± 0.21 g/L), folic acid (2.86 ± 0.25 g/L), calcium pantothenate (2.80 ± 0.35 g/L), *p*-aminobenzoic acid (2.63 ± 0.26 g/L), biotin (2.61 ± 0.21 g/L), thiamine hydrochloride (2.48 ± 0.35 g/L), niacin (2.24 ± 0.26 g/L), pyridoxine hydrochloride (2.18 ± 0.08 g/L), and, finally, the YNB-VF (2.15 ± 0.23 g/L) (Table 1 and Figure 5A).

Biotin, folic acid, *p*-aminobenzoic acid, inositol, calcium pantothenate, thiamine hydrochloride, and YNB yielded the highest and similar maximum specific growth rates (0.14–0.17 1/h). The maximum volumetric biomass productivity (0.12–0.15 g/L·h) was achieved with biotin, calcium pantothenate, folic acid, *p*-aminobenzoic acid, inositol, and YNB (Table 1). In contrast, the lowest maximum specific growth rates (0.05–0.12 1/h) were observed with pyridoxine hydrochloride, niacin, and YNB-VF. Moreover, the lowest biomass productivities (0.05–0.10 g/L·h) were recorded with pyridoxine hydrochloride, thiamine hydrochloride, niacin, and YNB-VF.

Furthermore, vitamins did not significantly affect overall glucose consumption, as glucose was nearly exhausted in all yeast cultures, with overall glucose consumption efficiency ranging from 98.4% to 99.89% (Table 1 and Figure 5B) within 21–30 h of incubation. The exception was the culture supplemented with pyridoxine hydrochloride, where glucose depletion continued until 45–48 h of incubation.

Notably, the highest riboflavin production and volumetric productivity were achieved with the YNB-VF culture medium supplemented with biotin (9.62 ± 0.49 mg/L and 0.36 ± 0.002 mg/L·h, respectively) and the YNB medium (positive control) (8.88 ± 0.42 mg/L and 0.37 ± 0.08 mg/L·h, respectively) (Table 1 and Figure 5C,D), with no significant statistical difference between these two media (*p* > 0.05). However, there was a statistically significant difference in riboflavin production and volumetric productivity between the positive control (YNB) and biotin-supplemented culture compared to the negative control and cultures supplemented with the other vitamins (*p* < 0.05). *p*-Aminobenzoic acid and niacin also enhanced riboflavin production and productivity, but to a lesser extent than biotin. Specifically, riboflavin production and productivity with biotin were 80.8% and 89.47% higher, respectively, than with *p*-aminobenzoic acid, and 165.7% and 260% higher, respectively, than with niacin (Table 1 and Figure 5C,D).

In contrast, riboflavin production and volumetric productivity were the lowest in yeast cultures supplemented with calcium pantothenate, inositol, and thiamine hydrochloride, even falling below those of the negative control culture (YNB-VF). Specifically, riboflavin production in yeast cultures supplemented with pyridoxine hydrochloride, calcium pantothenate, and inositol was 14.8, 10.57, and 4.98 times lower, respectively, while volumetric productivity was 120, 12, and 7.2 times lower, respectively, than that obtained with biotin.

These results clearly indicate that biotin plays a crucial role in riboflavin biosynthesis by *H. wangnamkhiaoensis*. Therefore, biotin was selected for use in subsequent experiments. The YNB-VF medium supplemented with glucose, (NH_4_)_2_SO_4_, and biotin will henceforth be referred to as YNB-VF+B7.

Biotin is a water-soluble vitamin that acts as an essential coenzyme for several biotin-dependent enzymes, including carboxylases, decarboxylases, and transcarboxylases. These enzymes are found in all living organisms and catalyze important steps in several key metabolic pathways, such as fatty acid synthesis, gluconeogenesis, and amino acid catabolism, which are crucial for energy production and cell growth [59,60]. Additionally, biotin is vital for glucose catabolism and purine biosynthesis in *Saccharomyces cerevisiae* [49,61,62].

To the best of our knowledge, the precise metabolic role of biotin in riboflavin biosynthesis by micro-organisms remains undetermined. However, biotin is commonly included in many defined culture media used for microbial riboflavin production [33,45,58,63,64,65,66].

### 2.5. Formulation of a Culture Medium for Riboflavin Production by H. wangnamkhiaoensis in a Bubble Column Bioreactor

Although YNB, YNB-w/o-N, and YNB-VF are commonly used basal media for preparing minimal and synthetically defined culture media suitable for cultivating yeasts, they are expensive and contain chemical components prone to photo- and thermo-degradation. Consequently, these media are unsuitable for medium- and large-scale applications. Furthermore, these media contain iron, which strongly inhibits riboflavin biosynthesis [67,68].

Therefore, it is necessary to formulate a cost-effective culture medium that avoids the disadvantages of YNB, YNB-w/o-N, and YNB-VF culture media while remaining effective for riboflavin production by *H. wangnamkhiaoensis*. We proposed using Castañeda-Agulló’s (CA) culture medium as a basal medium for riboflavin production by *H. wangnamkhiaoensis* because it is inexpensive, has readily available components, and is highly suitable for cultivating yeasts, including *H. wangnamkhiaoensis* [17,34,35,38,69]. Kinetic studies were, therefore, conducted to assess riboflavin production by *H. wangnamkhiaoensis* in the CA medium and to compare the results obtained with those achieved using the previously optimized YNB-VF+B7 medium.

Kinetic experiments to assess cell growth, substrate consumption, and riboflavin production by *H. wangnamkhiaoensis* were performed in a bubble column bioreactor using the following culture media: (1) modified CA culture medium (MCA) supplemented with glucose (10 g/L) and (NH_4_)_2_SO_4_ (4.85 g/L) (MCA−B7); (2) MCA supplemented with glucose (10 g/L), (NH_4_)_2_SO_4_ (4.85 g/L), and biotin (20 μg/L) (MCA+B7); and (3) YNB-VF+B7 medium, which previously yielded the highest riboflavin production by *H. wangnamkhiaoensis*.

It is worth noting that the (NH_4_)_2_SO_4_ and KH_2_PO_4_ concentrations in the MCA−B7 and MCA+B7 culture media were adjusted to maintain the C/N and C/P ratios of 3.8 and 18.3, respectively, which are identical to the C/N and C/P ratios in the YNB-VF+B7 culture medium.

**Figure 6:**
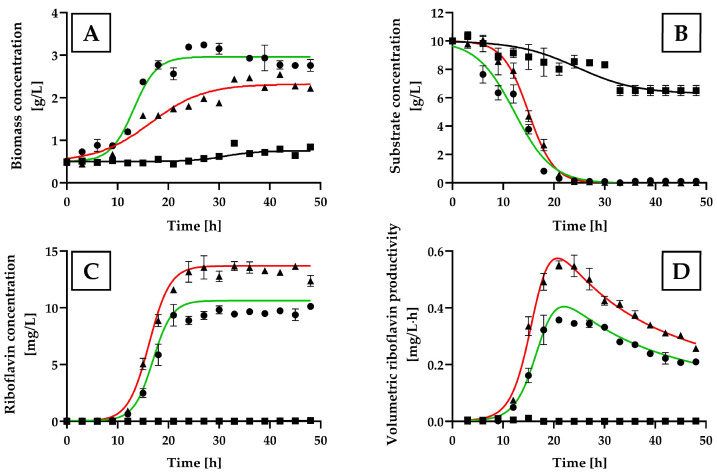
Comparison of the kinetic profiles of cell growth, glucose consumption, and riboflavin production by *Hyphopichia wangnamkhiaoensis* in various culture media. Experiments were conducted in a bubble column bioreactor using different culture media, represented by the following symbols: (●) YNB-VF+B7, (■) MCA−B7, and (▲) MCA+B7, at 28 ± 1 °C, pH 5.6, and 1 vvm of air flow rate. The kinetic profiles shown include (**A**) biomass concentration, (**B**) glucose concentration, (**C**) riboflavin concentration, and (**D**) volumetric riboflavin productivity. Error bars represent the standard deviation of independent triplicates. When the error bars are not shown, the standard deviation is smaller than the symbol size.

Figure 6 shows the kinetic profiles of cell growth, glucose consumption, and riboflavin production by *H. wangnamkhiaoensis* cultivated in different culture media.

*Hyphopichia wangnamkhiaoensis* exhibited the highest maximum specific growth rate (μ_max_ = 0.1744 ± 0.0129 1/h) and reached the highest biomass concentration (2.98 ± 0.24 g/L) when cultured in YNB-VF+B7 medium, followed by MCA+B7 medium (μ_max_ = 0.1371 ± 0.0070 1/h, 2.37 ± 0.14 g/L) (*p* < 0.05). In contrast, yeast growth in the MCA−B7 medium was significantly poorer (μ_max_ = 0.0263 ± 0.0026 1/h, 0.74 ± 0.09 g/L) (Figure 6A). These results indicate that adding a small amount of biotin to the MCA improved the maximum specific growth rate by 421.3% and the biomass concentration by 220.3% (*p* < 0.05).

The glucose consumption patterns were similar for yeast cultivated in YNB-VF+B7 and MCA+B7 media, with nearly complete glucose exhaustion occurring after approximately 24 h of incubation (98.92–99.93% glucose consumption efficiency). Conversely, the yeast grown in MCA−B7 consumed only approximately 34.96 ± 3.60% of the glucose during the first 33 h of incubation, with the residual glucose concentration remaining relatively constant thereafter (6.50 ± 0.36 g/L; Figure 6B).

Riboflavin production increased throughout the exponential growth phase of yeast cells cultivated in the YNB-VF+B7 medium. In contrast, yeast cells cultivated in the MCA+B7 medium produced riboflavin during the middle and late exponential growth phases (Figure 6C). As riboflavin is a growth-associated metabolite [66,67] in most flavinogenic micro-organisms, the highest riboflavin production was expected to occur in the YNB-VF+B7 medium because *H. wangnamkhiaoensis* exhibited a higher maximum specific growth rate (μ_max_ = 0.1744 ± 0.0129 1/h) and produced a higher amount of biomass (2.98 ± 0.24 g/L) in the YNB-VF+B7 medium compared to the MCA+B7 medium (μ_max_ = 0.1371 ± 0.0070 1/h, 2.37 ± 0.14 g/L). However, yeast cells grown in the MCA+B7 medium produced 37.6% more riboflavin (13.24 ± 0.61 mg/L) than those grown in the YNB-VF+B7 medium (9.62 ± 0.49 mg/L) (*p* < 0.05). Furthermore, yeast produced negligible amounts of riboflavin when grown in the MCA−B7 medium (0.05 ± 0.01 mg/L) (Figure 6C).

When YNB-VF+B7 and MCA+B7 were used as culture media, peaks of volumetric riboflavin productivity were reached at 21 h of incubation, with values of 0.36 ± 0.002 and 0.55 ± 0.01 mg/L·h, respectively, showing a statistically significant difference between them (*p* < 0.05). Therefore, the maximum volumetric productivity of riboflavin in the MCA+B7 medium was 52% higher than that in the YNB-VF+B7 medium (*p* < 0.05). Furthermore, the volumetric productivity of riboflavin in *H. wangnamkhiaoensis* grown in MCA−B7 medium was negligible.

Similarly, when the YNB-VF+B7, MCA+B7, and MCA−B7 culture media were used, the maximum riboflavin yields based on consumed glucose (Y_PS_) were 0.97 ± 0.05, 1.32 ± 0.06, and 0.02 ± 0.001 mg of riboflavin/g of glucose, whereas the maximum biomass yields based on consumed glucose (Y_XS_) were 0.26 ± 0.02, 0.19 ± 0.01, and 0.09 ± 0.04 g of biomass/g of glucose, respectively.

These results indicate that the best culture medium for riboflavin production by *H. wangnamkhiaoensis* is the MCA+B7 medium, as it achieved the highest concentration, yield, and volumetric productivity of riboflavin. In contrast, YNB-VF+B7 was found to be more suitable for biomass production, as it resulted in the highest biomass concentration and yield compared to MCA−B7 and MCA+B7.

Additionally, glycine, threonine, and serine have been shown to enhance riboflavin production in several micro-organisms [1,2,6,32,33,45,63,70,71,72,73]. Considering these findings, the influence of these amino acids, which are absent in YNB, YNB-w/o-N, and YNB-VF, on riboflavin production by *H. wangnamkhiaoensis* was further investigated. For this purpose, the MCA+B7 medium was supplemented with L-serine, glycine, or DL-threonine at 15 mM, while the MCA+B7 medium without amino acids was used as a control medium. It is worth mentioning that the (NH_4_)_2_SO_4_ and KH_2_PO_4_ concentrations of the MCA+B7 medium supplemented with an amino acid (glycine, L-serine, and DL-threonine) were adjusted to maintain the C/N and C/P ratios at 3.8 and 18.3, respectively.

**Figure 7:**
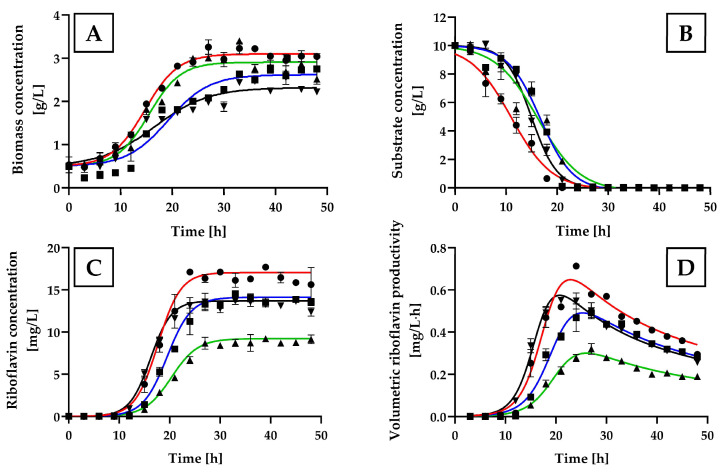
Effect of glycine, L-serine, and DL-threonine on the kinetics of *Hyphopichia wangnamkhiaoensis* cultures. Kinetic profiles of cell growth, glucose consumption, and riboflavin production by *H. wangnamkhiaoensis* in MCA+B7 control culture medium (▼) and MCA+B7 medium supplemented with (●) glycine, (■) L-serine or (▲) DL-threonine, in a bubble column reactor at 28 ± 1 °C, pH 5.6, and 1 vvm of air flow rate. The kinetic profiles shown include (**A**) biomass concentration, (**B**) glucose concentration, (**C**) riboflavin concentration, and (**D**) volumetric riboflavin productivity. Error bars represent the standard deviation of independent triplicates. When the error bars are not shown, the standard deviation is smaller than the symbol size.

The addition of glycine, L-serine, or DL-threonine to the MCA+B7 medium significantly increased the biomass production of *H. wangnamkhiaoensis* (Figure 7A), with the highest values observed for glycine (3.07 ± 0.17 g/L), followed by DL-threonine (2.89 ± 0.27 g/L), L-serine (2.66 ± 0.16 g/L), and the control culture (MCA+B7 without amino acids) (2.37 ± 0.14 g/L). Additionally, the maximum specific growth rates obtained with glycine, L-serine, DL-threonine, and the control culture were 0.1368 ± 0.0055, 0.1248 ± 0.0081, 0.1089 ± 0.0055, and 0.1371 ± 0.0070 1/h, respectively. The glucose consumption patterns were consistent with the cell growth patterns (Figure 7B), with all the glucose initially present in the culture medium being exhausted within 21–30 h of incubation.

The highest levels of riboflavin production and volumetric productivity were achieved between approximately 21 and 27 h of incubation, which coincided with the middle and late exponential growth phases of *H. wangnamkhiaoensis* (Figure 7A,C,D). The yeast culture supplemented with glycine showed significantly higher riboflavin production and riboflavin productivity compared to those supplemented with other amino acids and the control culture (*p* < 0.05) (Figure 7C,D), with the values of 16.68 ± 0.69 mg/L and 0.7128 ± 0.01 mg/L·h, respectively, which were 21.04% and 45%, 91.94% and 122%, and 25.98% and 22.7% higher than those obtained with L-serine, DL-threonine and the control culture, respectively.

Glycine also enhanced riboflavin production in *Candida famata* and *Ashbya gossypii* [1,2,6,33,45,70], whereas threonine and serine were effective in *Eremothecium ashbyii* [6,63,72]. Glycine metabolism is closely related to that of serine and threonine in yeasts [6,55,56,74,75]. The differences in their effectiveness in riboflavin biosynthesis lie in their amino acid uptake [1] and the specific activity of threonine aldolase or serine hydroxymethyltransferase, enzymes that catalyze the synthesis of glycine from threonine or serine, respectively [32,74,75].

Based on the above results, glycine was selected as a constituent in formulating an appropriate and effective culture medium for riboflavin production by *H. wangnamkhiaoensis*, designated as the RGE culture medium.

The RGE culture medium contains the following low-cost and readily available chemical components: dibasic ammonium citrate (0.625 g/L), KH_2_PO_4_ (1.0942 g/L), MgSO_4_·7H_2_O (0.275 g/L), Na_2_CO_3_ (0.375 g/L), NaCl (0.250 g/L), glucose (10 g/L), (NH_4_)_2_SO_4_ (4.3037 g/L), biotin (0.02 mg/L), and glycine (1.1261 g/L).

A notable aspect is that the maximum production and volumetric productivity of riboflavin by *H. wangnamkhiaoensis* are achieved at approximately 18–24 h of incubation. In contrast, the wild-type and recombinant strains of *C. famata* and *Meyerozyma guilliermondii* require approximately 72–240 h of incubation to reach maximum riboflavin production levels [2,6,20,47].

The RGE culture medium offers a cost-effective alternative for riboflavin production by *H. wangnamkhiaoensis*. Producing 1 g of riboflavin with RGE medium costs approximately USD 243.94, compared to USD 1548.22 with YNB and USD 456.41 with YNB-VF+B7, using analytical-grade reagents for all media. Furthermore, 1 m^3^ of RGE medium costs USD 4064.24, whereas YNB and YNB-VF+B7 cost USD 13,727.64 and 4381.11, respectively.

Further investigation into growth and riboflavin production conditions is necessary as they may significantly impact outcomes. Our findings support the development of cost-effective methods for riboflavin production.

## 3. Materials and Methods

### 3.1. Micro-Organism

The *H. wangnamkhiaoensis* ENCB-7 yeast strain used in this study was obtained from the Industrial Microbiology Laboratory Culture Collection of the National School of Biological Sciences at the National Polytechnic Institute (ENCB-IPN), Mexico City, Mexico. This yeast species was preserved on yeast nitrogen base (YNB)-glucose agar plates and slants with the following formulation: 6.7 g/L YNB (Becton, Dickinson and Company, Sparks, MD, USA), 10 g/L glucose (Sigma-Aldrich, Co., Ltd., Santa Clara, CA, USA), and 20 g/L bacteriological agar (Becton, Dickinson and Company, Franklin Lakes, NJ, USA).

### 3.2. Culture Media

In this study, we used YNB, YNB without amino acids and ammonium sulfate (YNB-w/o-N), and YNB without vitamins (YNB-VF). The first two culture media were purchased from BD Difco^TM^ (Becton Dickinson and Company), and the last one was purchased from Formedium Ltd. (Hunstanton, Norfolk, UK). The composition of these media is reported in the Kurtzman’s Yeast Manual [76] and is shown in Table 2.

To formulate a cost-effective culture medium for riboflavin production by *H. wangnamkhiaoensis*, we evaluated Castañeda-Agulló’s (CA) culture medium as basal medium for yeast cultivation. The CA medium contains 0.625 g/L dibasic ammonium citrate, 0.375 g/L KH_2_PO_4_, 0.275 g/L MgSO_4_·7H_2_O, 0.375 g/L Na_2_CO_3_, and 0.250 g/L NaCl [77]. All these chemical components were purchased from JT Baker (Avantor Performance Materials, Inc., Xalostoc, Estado de México, Mexico).

### 3.3. Pneumatic Bubble Column Bioreactor

**Figure 8:**
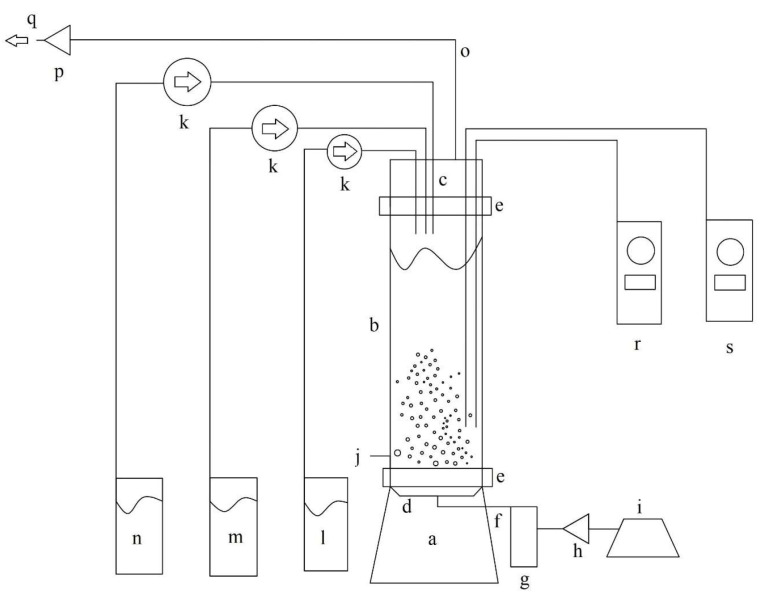
Schematic representation of bubble column bioreactor. The components of a bubble column reactor include the following: (a) base, (b) cylindrical column, (c) lid, (d) air diffuser, (e) Nylamid flanges, (f) sterile air input, (g) flowmeter, (h) air filter, (i) diaphragm pump, (j) sampling port, (k) peristaltic pumps, (l) acid reservoir, (m) alkali reservoir, (n) antifoam reservoir, (o) exhaust airline, (p) exhaust air filter, (q) exhaust air outlet, (r) pH controller, and (s) temperature controller.

Bubble column bioreactors, which create a relatively low shear stress environment, are particularly suitable for cultivating shear stress-sensitive micro-organisms. Additionally, bubble column bioreactors require less power compared to mechanically agitated bioreactors and are among the most cost-effective designs for producing industrially valuable products [78,79]. Thus, a bubble column bioreactor was employed in this study to minimize cell damage, enhance riboflavin production, and reduce production costs. Figure 8 shows a schematic diagram of the bubble column bioreactor.

The bubble column bioreactor has a total volume of 600 mL and a working volume of 450 mL. It is composed of three borosilicate glass components: (1) a base with a centered glass-fiber porous diffuser, hermetically sealed and connected to the sterile air input port, (2) a cylindrical column, and (3) a lid with ports for adding the culture medium, alkali, acid, antifoam, and an exhaust air outlet port. The cylindrical column is joined to the base and lid using stainless steel screws that connect Nylamid flanges, fastening the glass lips of the column, base, and lid between two Nylamid flanges. Sterile neoprene O-rings lubricated with silicone-based thermal grease were placed between two glass lips to prevent glass-to-glass contact and to hermetically seal the bioreactor junctions. The airflow rate is controlled using a pressure-regulating valve and measured using a calibrated rotameter.

### 3.4. Kinetic Study of the Effect of Different Nutritional Factors on Riboflavin Production by H. wangnamkhiaoensis

Batch culture experiments were conducted in a bubble column bioreactor to determine the effects of different nutritional factors on the growth, substrate consumption, and riboflavin production in *H. wangnamkhiaoensis*. The effects of different carbon and energy sources, nitrogen sources, amino acids, and vitamins were investigated using YNB, YNB-w/o-N, YNB-w/o-N, and YNB-VF as basal media, respectively. These culture media were initially used as basal media because previous studies have shown that *H. wangnamkhiaoensis* can produce riboflavin in these media.

Solutions of the basal culture media (YNB, YNB-w/o-N, and YNB-VF) and vitamins were sterilized by microfiltration through mixed cellulose ester Millipore^®^ membranes (Merck KGaA, Darmstadt, Germany) with a pore size of 0.22 μm. In contrast, solutions of carbon sources, nitrogen sources, and amino acids were sterilized by autoclaving at 121 °C for 15 min.

To investigate the effect of nutritional factors, the inocula were prepared by placing an inoculation loop of *H. wangnamkhiaoensis* in Erlenmeyer flasks containing culture media with the same chemical composition as those used in the kinetic experiments performed in the bubble column bioreactor. The flasks were agitated at 120 rpm at 28 ± 1 °C for 12 h. Afterward, the cell suspensions were centrifuged at 3500 rpm for 15 min to separate the yeast biomass, which was washed twice with sterile distilled water. The biomass was then resuspended in a small volume of culture medium for testing and the resulting cell suspension was homogenized. A biomass suspension sample was used to inoculate the bioreactor.

Batch cultures in the bioreactor were conducted for 48 h, at 28 ± 1 °C, pH 5.6, with an initial yeast biomass concentration of 0.5 ± 0.06 g/L, and using YNB, YNB-w/o-N, or YNB-VF as basal media, which were supplemented with a carbon source, a nitrogen source, and a vitamin and/or an amino acid.

The airflow rate supplied to the bioreactor was 1 vvm, which was selected in previous studies because it improved the biomass and riboflavin production by *H. wangnamkhiaoensis* [37,69]. Yeast culture samples were collected every 3 h to determine the biomass growth, substrate consumption, and riboflavin production.

#### 3.4.1. Effect of Different Carbon Sources

The influence of the carbon source on riboflavin production was assessed using 6.7 g/L YNB basal medium supplemented with 10 g/L glucose, maltose, starch, or glycerol. The carbon source that yielded the highest riboflavin production was selected for further experiments.

#### 3.4.2. Influence of Different Nitrogen Sources

The effect of the nitrogen source on kinetic performance was examined using YNB-w/o-N basal medium at a concentration of 1.7 g/L, which was supplemented with 5 g/L of the nitrogen source to be tested and 10 g/L of the previously selected carbon source. The nitrogen sources assayed included DL-glutamic acid, L-glutamic acid, urea (CH_4_N_2_O), ammonium acetate (NH_4_CH_3_CO_2_), ammonium sulfate [(NH_4_)_2_SO_4_], diammonium phosphate [(NH_4_)_2_HPO_4_], ammonium chloride (NH_4_Cl), sodium nitrate (NaNO_3_), potassium nitrate (KNO_3_), and sodium nitrite (NaNO_2_). Initially, a preliminary screening of nitrogen sources was conducted at the flask level, and those showing the best performance for riboflavin production were further tested in a bubble column bioreactor. The nitrogen source that showed the best riboflavin production characteristics in the bubble column bioreactor was selected for subsequent studies.

#### 3.4.3. Effect of Some Amino Acids

L-histidine, DL-methionine, and DL-tryptophan are amino acid constituents of the YNB medium; therefore, in this study, we investigated whether they had any effect on riboflavin production by *H. wangnamkhiaoensis*. These studies were performed using 1.7 g/L YNB-w/o-N supplemented with 10 g/L and 5 g/L of the previously selected carbon and nitrogen sources, respectively, and 10 mg/L L-histidine, 20 mg/L DL-methionine, or 20 mg/L DL-tryptophan. The tested concentrations of amino acids were those in the YNB medium (Table 2).

#### 3.4.4. Influence of Some Vitamins

The effect of the vitamin constituents of YNB medium, except for riboflavin, on riboflavin production by *H. wangnamkhiaoensis* was explored. These studies were conducted using 16.7 g/L of YNB-VF medium containing 10 g/L of glucose and 5 g/L of ammonium sulfate, and supplemented with 20 μg/L of biotin, 2000 μg/L of calcium pantothenate, 2 μg/L of folic acid, 10,000 μg/L of inositol, 400 μg/L of niacin, 200 μg/L of *p*-aminobenzoic acid (*p*ABA), 400 μg/L of pyridoxine hydrochloride, or 400 μg/L of thiamine hydrochloride. The tested concentrations of vitamins were those in the YNB medium.

The culture medium with the highest riboflavin production was designated YNB-VF+B7.

### 3.5. Formulation of a Culture Medium for Riboflavin Production by H. wangnamkhiaoensis

To formulate a culture medium of lower cost than the YNB, YNB-w/o-N, and YNB-VF media and that is effective for riboflavin production by *H. wangnamkhiaoensis*, the Castañeda-Agulló’s (CA) culture medium was evaluated and selected as the basal medium because it is a low-cost and suitable medium for the cultivation of yeasts, and its ingredients are readily available.

The carbon source, the nitrogen source, vitamin, and/or amino acid previously selected were added to the Castañeda-Agulló’s (CA) medium. The original concentration of monobasic potassium phosphate in the CA medium was modified such that the resulting culture medium (MCA) had a C/P ratio of 18.3, and the MCA had a C/N ratio of 3.8. Kinetic studies of cell growth, carbon source consumption, and riboflavin production were carried out in batch cultures using MCA with a pH of 5.6, and the results obtained were compared with those obtained previously using YNB-VF+B7 medium.

Furthermore, several studies have shown that glycine, threonine, and serine enhance riboflavin production in various micro-organisms [1,2,6,32,33,45,63,70,71,72,73]. Therefore, the present study investigated the effects of these amino acids on riboflavin production in *H. wangnamkhiaoensis*. These experiments were performed in MCA, which was supplemented with the previously selected carbon source, nitrogen source, amino acids (histidine, methionine, or tryptophan), and vitamins, with the addition of glycine, DL-threonine, or L-serine at 15 mM [73]. The resulting culture media had C/N and C/P ratios of 3.8 and 18.3, respectively, and a pH of 5.6.

### 3.6. Analytical Methods

#### 3.6.1. Cell Concentration

The yeast biomass concentration was measured gravimetrically using the dry cell weight method, following the procedures outlined by Chávez-Camarillo et al. [17]. Briefly, yeast culture samples were filtered through pre-weighed 1.6 μm microfiber filters (Whatman GF/A) (Cytiva, St. Louis, MO, USA), and the biomass retained was washed twice with sterile distilled water and subsequently dried at 60 °C until reaching a constant weight. Biomass concentration was quantified by subtracting the filter weight before filtration from that after filtration and drying. The filtrates were used to determine substrate and riboflavin concentrations.

#### 3.6.2. Substrate Concentration

Glucose, maltose, and starch concentrations were estimated using the glucose oxidase-peroxidase [34], dinitrosalicylic acid [80], and anthrone [35,81] methods, respectively. The glycerol concentration was determined according to the procedure proposed by Bondioli and Della Bella [82]. The glucose, maltose, starch, and glycerol concentrations were proportional to their absorbance and quantified using external standards with 11-point calibration curves.

#### 3.6.3. Riboflavin Concentration

The riboflavin concentration was measured using a SpectraMax M3 fluorometer (Molecular Devices LCC, San Jose, CA, USA) at excitation and emission wavelengths of 450 and 525 nm, respectively [38]. Riboflavin concentrations were proportional to their relative fluorescence units (RFU) and were quantified using an external riboflavin standard (Supelco Inc., Bellefonte, PA, USA) with an 11-point calibration curve.

### 3.7. Data and Statistical Analysis

All experiments were conducted in triplicates. The kinetic data on the *H. wangnamkhiaoensis* performance are the average of at least 6 assays (*n* ≥ 6). Data were statistically evaluated with one-way ANOVA using Bonferroni’s multiple comparison post hoc test and GraphPad Prism version 8.02 (GraphPad Software, Boston, MA, USA), with *p* < 0.05. Results are expressed as mean value ± standard deviation of independent triplicates.

## 4. Conclusions

To our knowledge, this study is the first to perform kinetic studies of the effects of various carbon and energy sources, nitrogen sources, vitamins, and amino acids on riboflavin production by *H. wangnamkhiaoensis* in a pneumatic bubble column bioreactor. The highest riboflavin production was achieved when glucose, ammonium sulfate, biotin, and glycine were added to the yeast growth medium. A novel, low-cost, and effective culture medium, called RGE medium, was formulated using these chemical components along with those in Castañeda-Agullo’s culture medium, which led to riboflavin production at a concentration of 16.68 mg/L and a volumetric productivity of 0.713 mg/L·h. Ongoing studies aim to further improve riboflavin production by *H. wangnamkhiaoensis* using different reaction systems.

## Figures and Tables

**Table 1:** Effects of different vitamins on the kinetic parameters of cell growth, glucose consumption, and riboflavin production by *Hyphopichia wangnamkhiaoensis*.

	ΔX_max_ (g/L)	μ_max_ (1/h)	Rx_max_ (g/L·h)	E (%)	P (mg/L)	Rp_max_ (mg/L·h)
Positive control (YNB)	2.91 ± 0.21	0.16 ± 0.02	0.15 ± 0.01	99.29 ± 0.19	8.88 ± 0.42	0.37 ± 0.08
Negative control (YNB-VF)	2.15 ± 0.23	0.12 ± 0.03	0.07 ± 0.01	99.55 ± 0.10	2.28 ± 0.16	0.07 ± 0.01
Biotin	2.61 ± 0.21	0.17 ± 0.01	0.13 ± 0.01	98.92 ± 0.10	9.62 ± 0.49	0.36 ± 0.001
Calcium pantothenate	2.80 ± 0.35	0.14 ± 0.01	0.14 ± 0.01	99.51 ± 0.01	0.91 ± 0.08	0.03 ± 0.001
Folic acid	2.86 ± 0.25	0.17 ± 0.02	0.14 ± 0.01	98.40 ± 0.09	2.70 ± 0.33	0.11 ± 0.01
Inositol	3.24 ± 0.20	0.16 ± 0.01	0.12 ± 0.01	99.65 ± 0.01	1.93 ± 0.08	0.05 ± 0.001
Niacin	2.24 ± 0.26	0.09 ± 0.01	0.10 ± 0.03	99.89 ± 0.01	3.62 ± 0.36	0.10 ± 0.02
*p*-Aminobenzoic acid	2.63 ± 0.26	0.17 ± 0.01	0.14 ± 0.01	98.48 ± 0.01	5.32 ± 0.24	0.19 ± 0.01
Pyridoxine hydrochloride	2.18 ± 0.08	0.05 ± 0.01	0.05 ± 0.01	99.74 ± 0.01	0.65 ± 0.16	0.003 ± 0.01
Thiamine hydrochloride	2.48 ± 0.35	0.17 ± 0.06	0.09 ± 0.01	99.74 ± 0.02	2.75 ± 0.38	0.10 ± 0.01

Batch cultures were performed in a bubble column bioreactor using yeast nitrogen base without vitamins (YNB-VF) medium at 28 ± 1 °C, pH 5.6, and 1 vvm of air flow rate. Results are expressed as mean value ± standard deviation of independent triplicates.

**Table 2:** Composition of YNB, YNB-w/o-N, and YNB-VF media.

	Component	YNB	YNB-w/o-N	YNB-VF
		Amount per Liter		
Carbon source	Glucose	0 g	0 g	10 g
Nitrogen source	Ammonium sulfate	5 g	0 g	5 g
Amino acids	L-histidine monohydrocloride	10 mg	0 mg	10 mg
	DL-methionine	20 mg	0 mg	20 mg
	DL-tryptophan	20 mg	0 mg	20 mg
Vitamins	Biotin	20 μg	20 μg	0 μg
	Calcium pantothenate	2000 μg	2000 μg	0 μg
	Folic acid	2 μg	2 μg	0 μg
	Inositol	10,000 μg	10,000 μg	0 μg
	Niacin	400 μg	400 μg	0 μg
	*p*-Aminobenzoic acid	200 μg	200 μg	0 μg
	Pyridoxine hydrochloride	400 μg	400 μg	0 μg
	Riboflavin	200 μg	200 μg	0 μg
	Thiamine hydrochloride	400 μg	400 μg	0 μg
Trace elements	Boric acid	500 μg	500 μg	500 μg
	Copper sulfate	40 μg	40 μg	40 μg
	Potassium iodide	100 μg	100 μg	100 μg
	Manganese sulfate	400 μg	400 μg	400 μg
	Ferric chloride	400 μg	400 μg	400 μg
	Sodium molybdate	200 μg	200 μg	200 μg
	Zinc sulfate	400 μg	400 μg	400 μg
Salts	Monobasic potassium phosphate	0.85 g	0.85 g	0.85 g
	Dibasic potassium phosphate	0.15 g	0.15 g	0.15 g
	Magnesium sulfate	0.5 g	0.5 g	0.5 g
	Sodium chloride	0.1 g	0.1 g	0.1 g
	Calcium chloride	0.1 g	0.1 g	0.1 g

## Data Availability

All relevant data are within the paper.
